# Re-description and dietary ecology of the *Hylaranaannamitica* (Sheridan & Stuart, 2018) (Amphibia: Ranidae) from central Vietnam

**DOI:** 10.3897/BDJ.13.e145094

**Published:** 2025-02-14

**Authors:** Duong Anh Thi To, Minh Duc Le, Hang Thu Thi Le, My Tra Phan, Anh Thi Ngoc Ho, Truong Quang Nguyen, Thomas Ziegler, Anh Van Pham

**Affiliations:** 1 Faculty of Environmental Sciences, University of Science, Vietnam National University, Hanoi, 334 Nguyen Trai Road, Hanoi, Vietnam Faculty of Environmental Sciences, University of Science, Vietnam National University, Hanoi, 334 Nguyen Trai Road Hanoi Vietnam; 2 Central Institute for Natural Resources and Environmental Studies, Vietnam National University, Hanoi, 19 Le Thanh Tong, Hanoi, Vietnam Central Institute for Natural Resources and Environmental Studies, Vietnam National University, Hanoi, 19 Le Thanh Tong Hanoi Vietnam; 3 Department of Herpetology, American Museum of Natural History, Central Park West at 79th Street, New York, Virgin Islands (USA) Department of Herpetology, American Museum of Natural History, Central Park West at 79th Street New York Virgin Islands (USA); 4 Institute of Ecology and Biological Resources, Vietnam Academy of Science and Technology, Hanoi, Vietnam Institute of Ecology and Biological Resources, Vietnam Academy of Science and Technology Hanoi Vietnam; 5 Graduate University of Science and Technology, VAST, Hanoi, Vietnam Graduate University of Science and Technology, VAST Hanoi Vietnam; 6 Cologne Zoo, Cologne, Germany Cologne Zoo Cologne Germany; 7 Institute of Zoology, University of Cologne, Cologne, Germany Institute of Zoology, University of Cologne Cologne Germany

**Keywords:** Invertebrates, prey items, stomach contents, vertebrates, Vu Quang National Park

## Abstract

**Background:**

The Annam Stream Frog *Hylaranaannamitica* was recently discovered from north and central Vietnam and Laos by Sheridan and Stuart (2018). Knowledge of its natural history is virtually lacking.

**New information:**

In this study, we provide an extended morphological description of *Hylaranaannamitica*, based on newly-collected specimens from Vu Quang National Park, Ha Tinh Province, Vietnam. In addition, we present data on the diet of *Hylaranaannamitica*, based on stomach content analyses of 46 individuals (32 males and 14 females) and compare prey selection between sexes. We found a total of 37 prey categories with 339 items, comprising 327 items of invertebrates, two items of vertebrates and 10 unidentified in the stomachs of *H.annamitica*. The most important (IRI) groups amongst the prey of *H.annamitica* were Coleoptera (17.19%), followed by Mantodea (14.78%), Orthoptera (11.26%), Lepidoptera (11.13%), Araneae (10.0%) and Blattodea (9.87%). There was an overlap of 45.63% in the diet between males and females and, in both sexes, the trophic spectrum was similar, predominantly consisting of Araneae, Coleoptera, Dermaptera, Diptera, Hemiptera, Hymenoptera, Isoptera, Lepidoptera, Mantodea and Orthoptera.

## Introduction

The diet composition can help to provide insights into the life history, population fluctuations and the impact of habitat modification on populations of anurans (Toft 1980, Donnelly 1991, Merlita and Olga 2013). Previous studies have shown that the diet of frogs depends on prey availability in the environment (Caldart et al. 2012, Merlita and Olga 2013, Ngo et al. 2014, Pham et al. 2019a, Pham et al. 2019b, Pham et al. 2024a). Therefore, identifying prey taxa for each species helps clarify the impact of frogs on invertebrate control (Nakamura and Tominaga 2021). However, detailed information on the diet of many frogs is either insufficient or lacking.

In Vietnam, the Annam Stream Frog *Hylaranaannamitica* (Sheridan & Stuart, 2018) is currently known from Bac Kan, Vinh Phuc, Thanh Hoa, Nghe An, Ha Tinh, Thua Thien - Hue, and Quang Nam provinces (Sheridan and Stuart 2018). Elsewhere, this species has been reported from Laos (Khammouan Province) (Sheridan and Stuart 2018). A recent preliminary study related to the diet of *H.annamitica* using 10 specimens collected from Ben En National Park (NP), Thanh Hoa Province, represents important progress towards the understanding of this amphibian species’ diet (Trinh et al. 2024). However, the small sample size in the analysis affects the generality of its conclusion.

Our study focuses on the Vu Quang NP, located in Ha Tinh Province, central Vietnam. This National Park was established in July 2002 by the Decision No. 102/QD-TTg of the Vietnam's Prime Minister and it covers an area of 55,029 hectares (Tordoff et al. 2004). Elevations at the Vu Quang NP range between 100 and 2,200 m. The highest points of the Park are found in the south along the mountain ridge that forms the international border between Vietnam and Laos (Tordoff et al. 2004). However, due to rough terrain, steep slopes and difficult access, surveys on the local amphibian fauna at this site are still limited.

In this study, we investigated the feeding ecology of *Hylaranaannamitica* in the Vu Quang NP, Vietnam. We examined: (1) its morphology; (2) its diet composition; (3) variation between sexes in prey composition. We also compared the results of this study with those reported from Ben En NP, Thanh Hoa Province.

## Materials and methods

**Sampling**

A field survey was conducted at three sites in Vu Quang NP, Ha Tinh Province, central Vietnam: compartment 155A within Vu Quang NP, from 20 to 29 April 2024 and compartment 165 within Vu Quang NP, from 30 April to 14 May 2024.

Frogs were found on the ground at edges of small slow-flowing streams or puddles and captured by hand along stream transects (approximately 2.0 - 3.0 km in length) between 19:30 and 23:30 h. The surrounding habitat was evergreen forest with shrubs. Air temperature was 20-26°C and relative humidity was 70-85% (The Vu Quang National Park 2017). We used a stomach-flushing technique to obtain stomach contents without sacrificing the frogs (Griffiths 1986, Leclerc and Courtois 1993, Solé et al. 2005). Spatula, forceps, two syringes with thread (60 ml) and the infusion tube of soft material (silicon) were used to obtain prey items from the stomach of frogs to avoid perforations of the oesophagus and stomach, in particular of small individuals. Each individual was stomach-flushed only once following the guidelines approved by the American Society of Ichthyologists and Herpetologists for animal care (Beaupre et al. 2004, Solé et al. 2005). The water used for flushing was taken from the streams where the frogs were captured and used after filtration. After stomach-flushing, frogs were monitored for vigour and body conditions and released within 30 minutes at the collecting sites. Prey items were preserved in 70% ethanol. We took measurements of snout-vent length (SVL), mouth width (MW) with a digital caliper to the nearest 0.1 mm and measured weight (BM) using electronic scales to the nearest 0.1 g.

**Species identification**

For taxonomic identification, two males and two females of the Annam Stream Frog were collected for voucher specimens. After having been photographed in life, animals were anaesthetised and euthanised in a closed vessel with a piece of cotton wool containing ethyl acetate (Simmons 2002), fixed in 85% ethanol and subsequently stored in 70% ethanol. Measurements were taken with a digital calliper to the nearest 0.1 mm. Abbreviations are as follows: a.s.l.: above sea level; terminology of morphological characters followed Sheridan and Stuart (2018): snout-vent length (SVL), head length from tip of snout to rear of jaws (HDL), mouth width (MW), snout length from tip of snout to anterior corner of eye (SNT), eye diameter (EYE), interorbital distance (IOD), internasal distance (IND), horizontal diameter of tympanum (TMP), shank length (SHK), thigh length (TGH), manus length from tip of third digit to base of outer palmar tubercle (HND) and pes length from tip of fourth toe to base of inner metatarsal tubercle (FTL).

Determination of species, based on morphology, followed Sheridan and Stuart (2018). In addition, we sequenced one new sample of *Hylaranaannamitica* collected from Ha Tinh Province. We used the protocols of Le et al. (2006) for DNA extraction, amplification and sequencing. A fragment of the mitochondrial gene, 16S ribosomal RNA, approximately 560 bp was amplified and sequenced using a primer pair AR (5’- CGCCTGTTTATCAAAAACAT - 3’) and BR (5’- CCGGTCTGAACTCAGATCACGT - 3’) (Palumbi et al. 1991). Sequences were compared with those from GenBank using Basic Local Alignment Search Tool (BLAST) searches.

**Stomach content analysis**

Prey items were identified under a microscope (Olympus SZ 700), based on identification keys (Naumann et al. 1991, Johnson and Triplehorn 2005, Brusca et al. 2016, Thai 2022). The maximum length (L) and width (W) of each prey item were measured to the nearest 0.1 mm using either a caliper or a calibrated ocular micrometer fitted to a microscope. Bodied parts of the same individual were assembled before taking measurements, otherwise incomplete-bodied prey were measured separately. The volume (V) of prey item was calculated using the formula for a prolate spheroid (*π* = 3.14; Magnusson et al. (2003)): \begin{varwidth}{50in}\begin{equation*}
            V=4π/3*(L/2)*(W/2)^2
        \end{equation*}\end{varwidth}.

To evaluate the relative importance of each prey category, we calculated the following three indices: %F, the frequency of occurrence (the percentage of stomachs containing specific prey categories amongst stomachs containing prey categories); %N, the relative number (the percentage of a specific prey categories amongst the number of the bulk of prey categories); and %V, the relative volume (the percentage of the volume of a specific prey categories amongst the volume of the bulk of prey categories (Nakamura and Tominaga 2021).

The index of relative importance (IRI) was used to determine the importance of each food category. This index provides a more informed estimation of prey item consumption than any of the three components alone by using the following formula (Krebs 1999, Pham and Nguyen 2018, Pham et al. 2019a, Pham et al. 2019b, Pham et al. 2022, Pham et al. 2023, Pham et al. 2024a, Pham et al. 2024b): IRI = (%F+%N+%V)/3,

where F is the frequency of prey occurrence in stomachs and N is the total number of prey items concerning all prey items.

We used the reciprocal Simpson’s heterogeneity index, 1-D, to calculate dietary heterogeneity: \begin{varwidth}{50in}\begin{equation*}
            D = ∑[n (n -- 1)]/[N(N -- 1)]
        \end{equation*}\end{varwidth}, where n is the number of prey items in the i taxon category and N is the total number of prey items (Pham et al. 2024a, Pham et al. 2024b).

To estimate prey evenness, we used Shannon’s Index of Evenness. Evenness is calculated from the equation: J’ = H’/H = H’/ln S. The maximum diversity (H) that could occur is that which would be found in a situation in which all taxa had equal abundance (H’ = H = ln S), S is the total number of prey taxa and H’ is the Shannon-Weiner index of taxon diversity. The value of H’ is calculated from the equation (Pham et al. 2024b): \begin{varwidth}{50in}\begin{equation*}
            H' = -∑P_i*ln(P_i)
        \end{equation*}\end{varwidth},

where *Pi* is the ratio of food items in the taxon to the total number of food items in the sample (Magurran 2004, Muñoz-Pedreros and Merino 2014).

We used linear regression to examine the relationship between mouth width (MW), snout-vent length (SVL), body mass (BM) and prey size. In addition, we determine the difference between sex.

To evaluate the relationships between the frog SVL and the prey volume of each individual, we calculated the following index values including minimum, maximum, mean prey item volume and total prey volume (Nakamura and Tominaga 2021).

Statistic analyses were performed using software package SPSS 20.0 (SPSS Inc., Chicago, Illinois, USA) and with the significance level set to P < 0.05 for all analyses. Data were presented as mean ± standard deviation (SD) unless otherwise noted. We used Kendall’s_tau b statistics to examine the number of prey items and prey volume from frogs of different sexes. We used one-way analysis of variance (ANOVA) to examine the size of prey items between sexes. The SVL and body mass (BW) of males and females were compared using a one-way ANOVA. Symbols: r is the correlation coefficient; the F_1_-value is an analysis of variance (ANOVA) test between two groups; the p-value represents the probability of obtaining a different result.

## Taxon treatments

### Hylarana annamitica

(Sheridan and Stuart, 2018)

4C151BF3-6EEF-56E7-A819-291FB71C0CFB

#### Materials

- **Type status:** Other material. **Occurrence:** catalogNumber: VQ.2024.10; individualCount: 1; sex: female; occurrenceID: 393818A1-4D65-5BB0-9CAA-850B3EADA599; **Taxon:** scientificNameID: *Hylaranaannamitica*; scientificName: *Hylaranaannamitica*; class: Amphibia; order: Anura; family: Ranidae; genus: Hylarana; specificEpithet: *annamitica*; scientificNameAuthorship: Sheridan & Stuart, 2018; **Location:** country: Vietnam; countryCode: VN; stateProvince: Ha Tinh; county: Vietnam; municipality: Vu Quang; locality: Vu Quang National Park; verbatimElevation: 310; verbatimLatitude: 18°17'31.3"N; verbatimLongitude: 105°21'04.8"E; verbatimCoordinateSystem: WGS84; **Event:** eventDate: April; eventTime: 2024; eventRemarks: collected by A.V. Pham and T.V. Bui; **Record Level:** language: en; collectionCode: Amphibia; basisOfRecord: PreservedSpecimen
- **Type status:** Other material. **Occurrence:** catalogNumber: VQ.2024.12; individualCount: 1; sex: male; occurrenceID: 2C23E6FE-2895-5D21-9CD4-C7E4F4AC6E97; **Taxon:** scientificNameID: *Hylaranaannamitica*; scientificName: *Hylaranaannamitica*; class: Amphibia; order: Anura; family: Ranidae; genus: Hylarana; specificEpithet: *annamitica*; scientificNameAuthorship: Sheridan & Stuart, 2018; **Location:** country: Vietnam; countryCode: VN; stateProvince: Ha Tinh; county: Vietnam; municipality: Vu Quang; locality: Vu Quang National Park; verbatimElevation: 310; verbatimLatitude: 18°17'31.3"N; verbatimLongitude: 105°21'04.8"E; verbatimCoordinateSystem: WGS84; **Event:** eventDate: April; eventTime: 2024; eventRemarks: collected by A.V. Pham and T.V. Bui; **Record Level:** language: en; collectionCode: Amphibia; basisOfRecord: PreservedSpecimen
- **Type status:** Other material. **Occurrence:** catalogNumber: VQ.2024.19; individualCount: 1; sex: female; occurrenceID: D5710AB1-B6F2-5C74-92C8-8D280709B7C0; **Taxon:** scientificNameID: *Hylaranaannamitica*; scientificName: *Hylaranaannamitica*; class: Amphibia; order: Anura; family: Ranidae; genus: Hylarana; specificEpithet: *annamitica*; scientificNameAuthorship: Sheridan & Stuart, 2018; **Location:** country: Vietnam; countryCode: VN; stateProvince: Ha Tinh; county: Vietnam; municipality: Vu Quang; locality: Vu Quang National Park; verbatimElevation: 310; verbatimLatitude: 18°17'31.3"N; verbatimLongitude: 105°21'04.8"E; verbatimCoordinateSystem: WGS84; **Event:** eventDate: April; eventTime: 2024; eventRemarks: collected by A.V. Pham and T.V. Bui; **Record Level:** language: en; collectionCode: Amphibia; basisOfRecord: PreservedSpecimen
- **Type status:** Other material. **Occurrence:** catalogNumber: VQ.2024.113; individualCount: 1; sex: male; occurrenceID: 065074F0-5F72-5B8B-9202-BC826C12CE3E; **Taxon:** scientificNameID: *Hylaranaannamitica*; scientificName: *Hylaranaannamitica*; class: Amphibia; order: Anura; family: Ranidae; genus: Hylarana; specificEpithet: *annamitica*; scientificNameAuthorship: Sheridan & Stuart, 2018; **Location:** country: Vietnam; countryCode: VN; stateProvince: Ha Tinh; county: Vietnam; municipality: Vu Quang; locality: Vu Quang National Park; verbatimElevation: 350; verbatimLatitude: 18°18'09.9"N; verbatimLongitude: 105°19'31.1"E; verbatimCoordinateSystem: WGS84; **Event:** eventDate: May; eventTime: 2024; eventRemarks: collected by A.V. Pham and T.V. Bui; **Record Level:** language: en; collectionCode: Amphibia; basisOfRecord: PreservedSpecimen

#### Description

A 563-bp long sequence (GenBank accession number PV075678) obtained from a specimen (Field No. VQ.2024.113) from Vu Quang NP was 99.82% similar to that with GenBank accession number AF285217 of *Hylaranaannamitica* collected in Ky Anh District, Ha Tinh Province.

**Figure 1.**
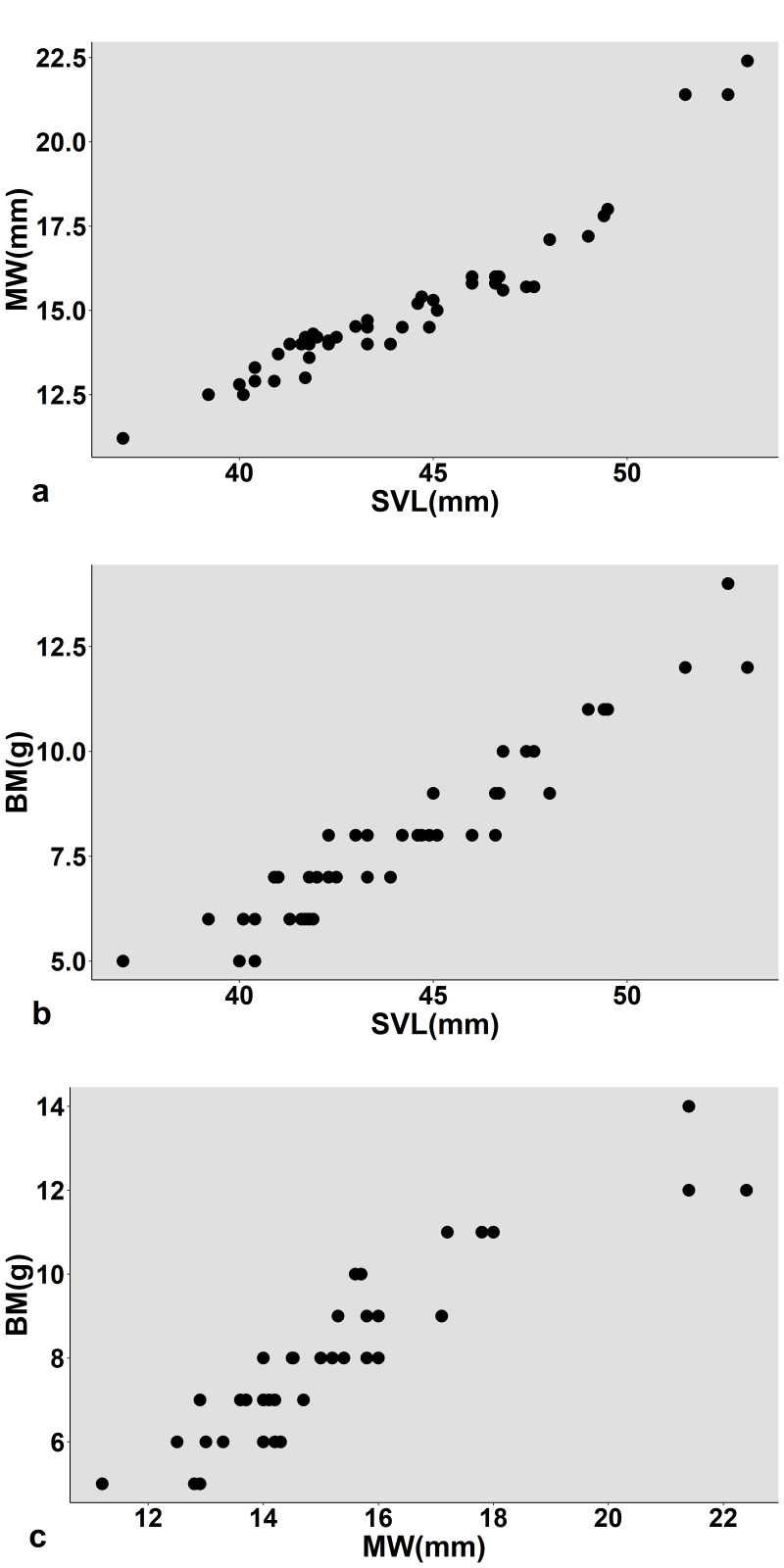
Dispersion diagrams from Pearson’s correlations between: (a) snout-vent length and mouth width, (b) snout-vent length and body mass and (c) mouth width and body mass of *Hylaranaannamitica* in Vu Quang NP, Ha Tinh Province, Vietnam

Morphological characteristics of specimens from Vietnam agreed well with the description of Sheridan and Stuart (2018): SVL min-max: 39.2–49.4 mm; mean and SD: 43.54 ± 2.76 mm, *n* = 32), MW 12.5-17.8 mm (14.56 ± 1.32 mm, *n* = 32) and body mass (BM 5.0–11.0 g, 7.50 ± 1.61 g, *n* = 32) in males was smaller than in females SVL 37.0–53.1 mm (46.10 ± 4.56 mm, *n* = 14); MW 11.2–22.4 mm (16.35 ± 3.34 mm, *n* = 14); BM 5.0–14.0 g, 9.07 ± 2.46 mm, *n* = 14). There were strong positive correlations between the morphological measurements (SVL and MW: *r* = 0.950, *P* < 0.001; SVL and BM: *r* = 0.949; *P* < 0.001; MW and BM: *r* = 0.907, *P* < 0.001; Fig. 1).

The following morphological characteristics were based on four preserved specimens. Snout obtusely pointed in dorsal view, rounded in profile; nostril closer to tip of snout than to eye; internarial distance greater than interorbital distance; eye diameter smaller than snout length; tympanum distinct, rounded, more than half eye diameter; vomerine teeth obliquely angled; tongue notched posteriorly; vocal sac openings near corner of jaw in males. Forelimbs: tips of fingers expanded into small discs with circummarginal grooves, relative finger lengths IV < II < I < III; webbing absent; two oval palmar tubercles; one oval thenar tubercle; humeral glands enlarged; nuptial pads small in males. Hind-limbs: tips of toes expanded into discs with circummarginal grooves; webbing present; inner metatarsal tubercle elongate; outer metatarsal tubercle round. See Table 1 for futher measurments.

Skin. Dorsal skin finely granular; distinct supratympanic fold; throat, chest, belly and ventral surface of thighs smooth; flanks slightly glandular; distinct dorsolateral folds.

**Figure 2.**
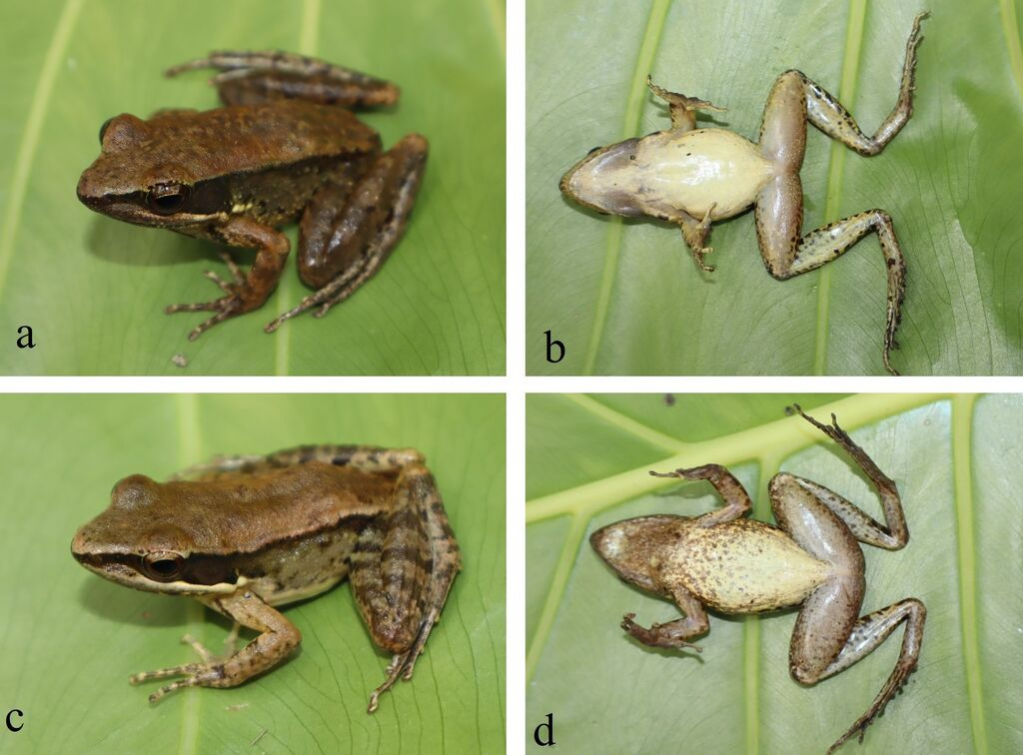
*Hylaranaannamitica* from Vu Quang NP, Ha Tinh Province, Vietnam: VQ.2024.113, adult male (a: dorsolateral view, b: ventral view); VQ.2024.10, adult female (c: dorsolateral view, d: ventral view).

Colouration in life. Dorsum reddish-brown, with some dark brown mottling spots; lip grey-brown anteriorly and white yellow posteriorly; dorsal surfaces of forelimbs and hind-limbs with dark crossbars; dorsolateral fold dark brown; flanks pale with dark brown spots; humeral glands dark brown; ventral surface slightly yellow or dark mottling on throat, less mottling on chest and underside of thighs (Fig. 2).

Ecology notes. This species was found on the ground near stream. Surrounding habitat was mixed evergreen forest of large hardwood, bamboo and shrub at elevations between 300 and 350 m.

#### Diet

*Prey items*

The number of prey items per individual was 1–44 (mean and SD: 7.37 ± 9.23 items, *n* = 46). The prey item length was 1.0–35.0 mm (min–max, n = 339); mean and prey item width was 0.2–17.0 mm (*n* = 339) in both sexes. The average dietary volume per individual was 1.31–5484.17 mm^3^ (*n* = 46).

**Figure 3.**
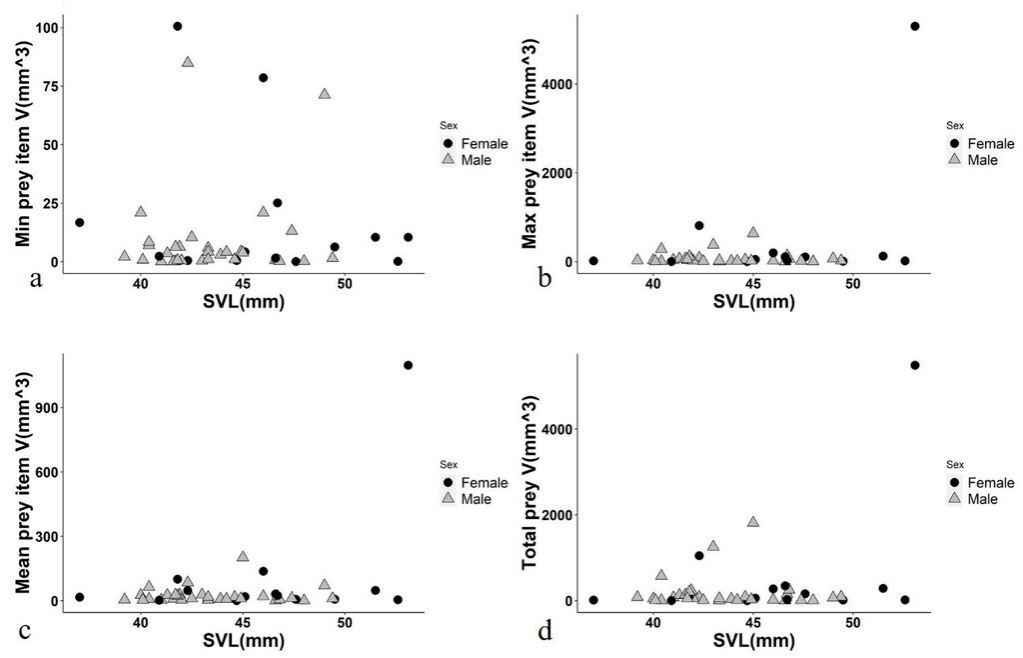
Relationships between the SVL of *Hylaranaannamitica* and the minimum (a), maximum (b) and the mean (c) prey item volume and the total prey volume (d). Open triangles: Males; Dots: Females.

There was no positive correlation between the SVL of frogs and the minimum prey volume (Kendall’s_tau b = 0.041, *P* = 0.682), maximum prey item volume (tau = 0.088, *P* = 0.37), the mean prey item volume (tau = 0.081, *P* = 0.407) and the total prey volume (tau = 0.089, *P* = 0.366) (Fig. 3). Similarly, no correlation for the minimum prey volume with the MW and BM of frogs (MW: tau = 0.040, *P* = 0.428, BM: tau = 0.033, *P* = 0.537), maximum prey item volume (MW: tau = 0.122 *P* = 0.218, BM: tau = 0.088, *P* = 0.391), the mean prey item volume (MW: tau = 0.115, *P* = 0.244, BM: tau = 0.065, *P* = 0.53) and the total prey volume (MW: tau = 0.136, *P* = 0.169, BM: tau = 0.095, *P* = 0.355) (Fig. 3).

*Dietary diversity*

We identified 37 prey categories in the stomachs of *Hylaranaannamitica* and other unidentified objects. Insects formed the main food component of *H.annamitica*, with 10 orders (Blattodae, Coleoptera, Dermaptera, Diptera, Hemiptera, Hymenoptera, Isoptera, Lepidoptera, Mantodea and Orthoptera) and insect larvae. Other categories included different invertebrate groups (Gastropoda, Araneae, Opiliones and Geophilomorpha) and Anura (Table 2). The highest proportion (%N) of prey items identified was Coleoptera (19.17%), followed by Blattodae (16.81%), Orthoptera (15.63%), Araneae (14.45%), Lepidoptera (6.19%) and Diptera (5.60%). In the most frequently foraged (%F) prey group was also Coleoptera (25.0%), followed by Araneae (12.50%), Lepidoptera and Orthoptera (10.23%) and Hymenoptera (6.82%). With regard to the IRI (%), Coleoptera (17.19%), followed by Mantodea (14.78%), Orthoptera (11.26%), Lepidoptera (11.13%), Araneae (10.0%) and Blattodae (9.87%) were found to be the most important prey groups (Table 2).

*Dietary differences between sexes*

**Figure 4.**
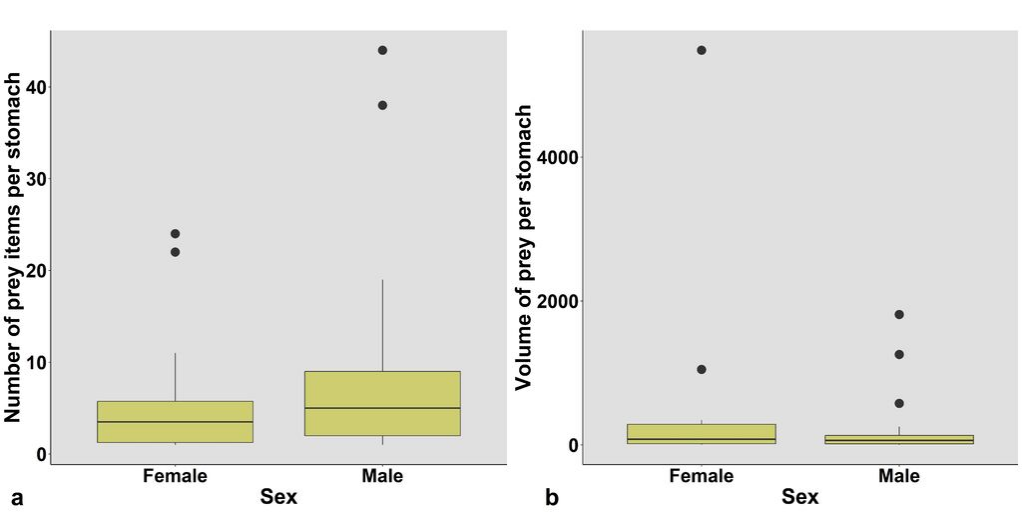
Boxplots representing factors that differed significantly amongst treatments: (a) Number of prey items of *Hylaranaannamitica* per stomach in male and female; (b) Prey volume per stomach in male and female.

The number of prey items found in males was 1–44 (7.88 ± 9.92 items, *n* = 32) and 1–24 in females (6.21 ± 7.62 items, *n* = 14) (Table 3), the values not being significantly different from each other (ANOVA, *F*_1, 44_ = 0.311, *P* = 0.58). The prey item length in males was 1.0–34.0 mm (*n* = 252) and 1.0–35.0 mm in females (*n* = 87), the values not being significantly different from each other (*F*_1, 337_ = 1.133, *P* = 0.288). The prey item width in males was 0.2–9.0 mm (*n* = 252) and 0.2–17.0 mm in females (*n* = 87). The values were also not significantly different between males and females (*F*_1, 337_ = 1.825, *P* = 0.178). The average dietary volume per individual in males was 1.31–1811.39 mm^3^ (*n* = 32) and 2.36–5484.17 mm^3^ (*n* = 14) in females, the values not being significantly different from each other (*F*_1_, _44_ = 1.963, *P* = 0.168) (Table 3, Fig. 4).

The Shannon–Wiener index of diet diversity in *Hylaranaannamitica* from Ha Tinh Province was *H* = 2.944. Adult males (25 prey categories, *H* = 2.619) consumed prey with slightly lower diversity than adult females (23 prey categories, *H* = 2.760).

**Figure 5.**
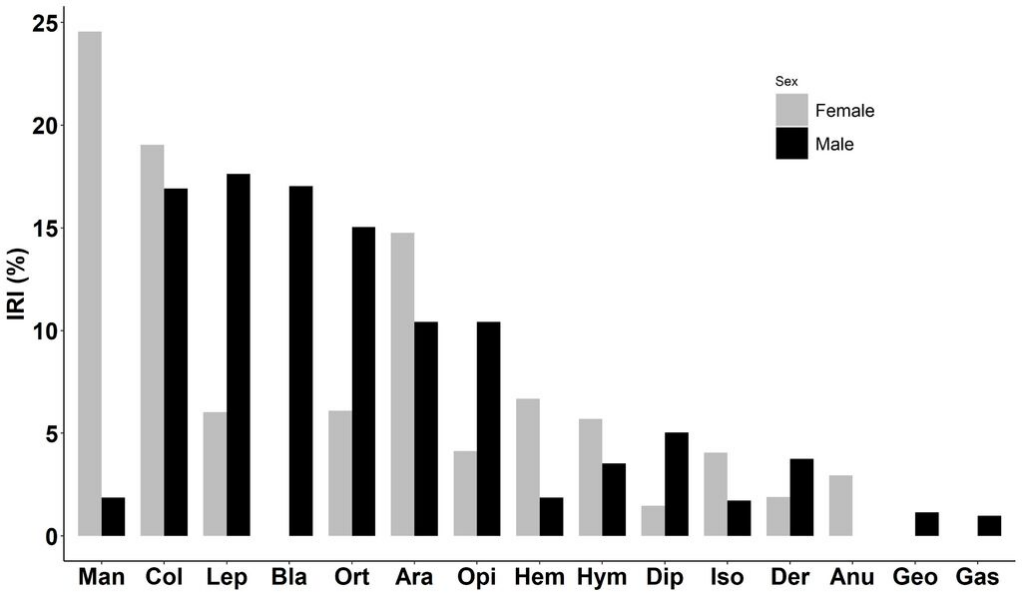
Importance indices (IRI) for prey categories consumed by males (black) vs. females (grey) of *Hylaranaannamitica* in Vietnam. Coleoptera (Col), Mantodea (Man), Araneae (Ara), Lepidoptera (Lep), Orthoptera (Ort), Blattodea (Bla), Opiliones (Opi), Hymenoptera (Hym), Hemiptera (Hem), Diptera (Dip), Isoptera (Iso), Dermaptera (Der), Anura (Anu), Geophilomorpha (Geo), Gastropoda (Gas).

There was an overlap of 45.63% in the diet between males and females. The trophic spectrum of males with the most important (IRI > 5%) consisted of Lepidoptera (17.62%), Blattodae (17.03%), Coleoptera (16.92%), Orthoptera (15.04%), Araneae (10.42%) and Diptera (5.03%), while the trophic spectrum of females with the most important (IRI > 5%) comprised Mantodea (24.56%), Coleoptera (19.05%), Araneae (10.62%), Hemiptera (6.67%), Orthoptera (6.10%), Lepidoptera (6.03%) and Hymenoptera (5.70%) (Fig. 5).

## Discussion

Trinh et al. (2024) recently reported the dietary composition of nine prey categories of *Hylaranaannamitica* in Ben En NP (*n* = 10). In this study, we recorded 28 additional prey categories of *Hylaranaannamitica* from Vietnam, including Pulmonata, Opiliones, Geophilidae, Blattidae, Anthicidae, Carabidae, Cerambycidae, Erotylidae, Eucnemidae, Nosodendridae, Psephenidae, Rhysodidae, Scarabaeidae, Anisolabididae, Pygidicranidae, Asilidae, Chironomidae, Culicidae, Tipulidae, Aradidae, Membracidae, Nabidae, Dryinidae, Ichneumonidae, Vespidae, Rhinotermitidae, Noctuidae, Mantidae, Acrididae, Gryllidae and Anura. However, we did not record prey items of Trochomorphidae, Lampyridae, Oedemeridae and Tettigoniidae reported in Trinh et al. (2024). Both studies show that major prey items of the species were Araneae, Pentatomidae and Formicidae (Suppl. material 1).

We found that Coleoptera form the main diet of *Hylaranaannamitica*. Coleoptera have also been reported as an important prey in the diet of many frogs probably because of its high availability in various tropical environments (Biavati et al. 2004, Wachlevski et al. 2008, Leavitt and Fitzgerald 2009, Quiroga et al. 2009, Oliveira et al. 2015, Pham and Nguyen 2018, Pham et al. 2019a, Pham et al. 2019b, Pham et al. 2022, Pham et al. 2023, Pham et al. 2024a, Pham et al. 2024b). In Vietnam, Coleoptera, Araneae, Orthoptera, Blattodea and Lepidoptera represent the most important prey categories (IRI > 5%) for *H.annamitica*, similar to the diet of *Limnonectesbannaensis*, *L. nguyenorum, Nanoranayunnanensis, Odorranachapaensis*, *Odorranajingdongensis* and *Polypedatesmegacephalus* (Pham and Nguyen 2018, Pham et al. 2019a, Pham et al. 2019b, Pham et al. 2023, Pham et al. 2024a). In addition, small anurans were also found in the stomach of one individual of *Hylaranaannamitica*. This prey has been documented in a few other frog species in Vietnam (Ngo et al. 2014, Pham and Nguyen 2018, Pham et al. 2019a, Pham et al. 2019b, Pham et al. 2024a, Pham et al. 2024b).

Pham et al. (2024b) documented the dietary composition of *Leptobrachellaeos*, another representative of the anurans also from Vu Quang NP, Vietnam. Both *Hylaranaannamitica* and *Leptobrachellaeos* inhabit similar streams. However, individuals of *H.annamitica* were found on the rocks or ground near water in the stream, while those of *L.eos* were spotted on the ground or tree branches, about 0.2–0.5 m above the forest floor (Pham et al. (2024b)). The prey categories (in taxon order of insect) of *H.annamitica* were more diverse than those in *L.eos* (15 vs. 12) (Pham et al. 2024b). Interestingly, diet content of *H.annamitica* is similar to that of *L.eos*, with Coleoptera, Blattodea, Orthoptera and Araneae being the most important prey items (IRI > 5%) (Pham et al. 2024b). Geophilomorpha, Diplura, Hymenoptera, Mantodea and Anura were detected exclusively in the diet of *Hylaranaannamitica*, whereas Decapoda and Plecoptera occurred only in the diet of *Leptobrachellaeos* (Pham et al. 2024b). The differences in the diet between *H.annamitica* and *L.eos* may reflect the differences in foraging strategy and microhabitat use.

In this study, we also found very small difference in the diet composition of males and females, as both sexes had a diverse prey spectrum, viz. Araneae, Coleoptera, Dermaptera, Diptera, Hemiptera, Hymenoptera, Isoptera, Lepidoptera, Mantodea and Orthoptera. Gastropoda and Blattodea were exclusively consumed by males, whereas Opiliones and Anura were only documented in the diet of females. The high similarities in diet between sexes may result from the fact that they occupy the same microhabitats. During our surveys, we often found males and females sympatrically. This phenomenon has also been reported in other frog species (Measey 1998, Biavati et al. 2004, Yu et al. 2009, Pamintuan and Starr 2016, Le et al. 2020).

## Supplementary Material

XML Treatment for Hylarana annamitica

2500438C-32BA-53A7-8B23-28E6BBBE7166 10.3897/BDJ.13.e145094.suppl1 Supplementary material 1 Dietary composition of *Hylaranaannamitica* in Vietnam Data type Table Brief description Dietary composition of *Hylaranaannamitica* in Vietnam. File: oo_1204412.docx https://binary.pensoft.net/file/1204412 To et al.

## Figures and Tables

**Table 1.** Measurements (in mm) of *Hylaranaannamitica* collected from Vu Quang NP, Ha Tinh Province, Vietnam.

	**VQ.2024.12**	**VQ.2024.113**	**VQ.2024.10**	**VQ.2024.19**	Sheridan and Stuart (2018)	
Sex	Male	Male	Female	Female	Males	Females
SVL	44.6	46.5	54.6	50.7	40.2–52.0	49.5–53.7
HDL	16.3	17.5	20.1	18.4	15.2–19.0	17.9–20.3
MW	15.5	16.3	18.7	17.4	13.6–17.9	16.3–18.4
SNT	6.2	6.4	7.6	6.9	5.8–7.5	6.9–7.6
EYE	5.5	5.6	7.0	6.5	5.3–6.8	6.3–7.1
IOD	3.6	3.8	4.7	4.3	3.2–5.4	4.2–4.8
IND	4.4	4.7	5.5	4.6	4.1–5.4	4.4–5.6
TMP	4.0	4.2	4.8	4.1	3.9–5.3	4.0–4.8
SHK	23.2	23.7	27.8	26.5	21.5–26.4	26.0–27.6
TGH	19.1	19.8	25.4	23.0	17.9–24.9	22.8–25.5
HND	12	12.7	14.2	13.2	10.0–12.7	12.2–13.7
FTL	22.3	22.9	27.4	24.1	20.1–26.3	24.0–27.3

**Table 2.** Prey categories consumed by *Hylaranaannamitica* in Vu Quang NP, Ha Tinh Province, Vietnam (n = 46), F) total frequency, %F) relative frequency, N) total abundance, %N) relative abundance, V) total volume (mm³), %V) relative volume; IRI) importance index.

Prey taxa	F	%F	N	%N	V	%V	IRI
**Gastropoda**	**1**	**1.14**	**1**	**0.29**	**41.89**	**0.31**	**0.58**
Pulmonata	1	1.14	1	0.29	41.89	0.31	0.58
**Arachnida**	**12**	**13.64**	**56**	**16.52**	**522.95**	**3.84**	**11.33**
Opiliones	1	1.14	7	2.06	106.11	0.78	1.33
Araneae	11	12.50	49	14.45	416.84	3.06	10.00
**Geophilomorpha**	**1**	**1.14**	**1**	**0.29**	**71.21**	**0.52**	**0.65**
Geophilidae	1	1.14	1	0.29	71.21	0.52	0.65
**Blattodea**	**2**	**2.27**	**57**	**16.81**	**1433.88**	**10.53**	**9.87**
Blattidae	2	2.27	57	16.81	1433.88	10.53	9.87
**Coleoptera**	**22**	**25.00**	**65**	**19.17**	**1008.43**	**7.40**	**17.19**
Carabidae	2	2.27	2	0.59	134.04	0.98	1.28
Cerambycidae	2	2.27	8	2.36	201.26	1.48	2.04
Curculionidae	1	1.14	2	0.59	2.09	0.02	0.58
Erotylidae	2	2.27	9	2.65	242.30	1.78	2.24
Eucnemidae	1	1.14	8	2.36	47.12	0.35	1.28
Nosodendridae	1	1.14	1	0.29	6.28	0.05	0.49
Psephenidae	1	1.14	3	0.88	12.30	0.09	0.70
Rhysodidae	1	1.14	2	0.59	12.04	0.09	0.60
Scarabaeidae	2	2.27	2	0.59	141.90	1.04	1.30
Larva	9	10.23	28	8.26	209.09	1.53	6.67
**Dermaptera**	**4**	**4.55**	**15**	**4.42**	**66.50**	**0.49**	**3.15**
Anisolabididae	3	3.41	14	4.13	39.27	0.29	2.61
Pygidicranidae	1	1.14	1	0.29	27.23	0.20	0.54
**Diptera**	**4**	**4.55**	**19**	**5.60**	**161.39**	**1.18**	**3.78**
Asilidae	1	1.14	11	3.24	124.74	0.92	1.77
Chironomidae	1	1.14	1	0.29	16.76	0.12	0.52
Culicidae	1	1.14	1	0.29	10.47	0.08	0.50
Tipulidae	1	1.14	6	1.77	9.42	0.07	0.99
**Hemiptera**	**4**	**4.55**	**6**	**1.77**	**869.53**	**6.38**	**4.23**
Aradidae	1	1.14	1	0.29	11.52	0.08	0.51
Membracidae	1	1.14	2	0.59	31.94	0.23	0.65
Nabidae	1	1.14	1	0.29	13.09	0.10	0.51
Pentatomidae	1	1.14	2	0.59	812.98	5.97	2.56
**Hymenoptera**	**6**	**6.82**	**15**	**4.42**	**108.38**	**0.80**	**4.01**
Dryinidae	1	1.14	8	2.36	44.77	0.33	1.27
Formicidae	2	2.27	2	0.59	25.13	0.18	1.02
Ichneumonidae	2	2.27	4	1.18	28.01	0.21	1.22
Vespidae	1	1.14	1	0.29	10.47	0.08	0.50
**Isoptera**	**3**	**3.41**	**12**	**3.54**	**59.36**	**0.44**	**2.46**
Rhinotermitidae	3	3.41	12	3.54	59.36	0.44	2.46
**Lepidoptera**	**9**	**10.23**	**21**	**6.19**	**2310.90**	**16.96**	**11.13**
Noctuidae	1	1.14	8	2.36	1800.92	13.22	5.57
Larva	7	7.95	11	3.24	474.51	3.48	4.89
Other Lepidoptera	1	1.14	2	0.59	35.47	0.26	0.66
**Mantodea**	**2**	**2.27**	**6**	**1.77**	**5489.93**	**40.30**	**14.78**
Mantidae	2	2.27	6	1.77	5489.93	40.30	14.78
**Orthoptera**	**9**	**10.23**	**53**	**15.63**	**1078.81**	**7.92**	**11.26**
Acrididae	7	7.95	36	10.62	344.46	2.53	7.03
Gryllidae	2	2.27	17	5.01	734.35	5.39	4.23
**Anura**	**1**	**1.14**	**2**	**0.59**	**274.89**	**2.02**	**1.25**
Unidentified	8	9.09	10	2.95	124.22	0.91	4.32

**Table 3.** Summary (Total, Mean, SD and range) of the prey item number (N), width (W), length (L) and volume (V) data for *Hylaranaannamitica* males and females (in mm for W and L; in mm^3^ for V).

	W	L	Prey item volume				N
			Minimum	Maximum	Mean	Total	
Male	1.72 ± 1.25	6.32 ± 5.19	9.02 ± 18.97	74.07 ± 129.80	24.66 ± 37.88	180.45 ± 378.64	7.88 ± 9.92
(*n* = 32)	0.2-9.0	1.0-34.0	0.05-84.82	0.65-636.17	0.65-201.27	1.31-1811.39	1-44
Female	1.9 9± 2.27	7.01 ± 5.34	18.40 ± 31.29	490.48 ± 1398.67	110.44 ± 286.67	560.56 ± 1443.73	6.21 ± 7.62
(*n* = 14)	0.2-17.0	1.0-35.0	0.08-100.53	1.05-5296.20	0.92-1096.83	2.36-5484.17	1-24
