# Supplementary material for: Re-description and dietary ecology of the Hylaranaannamitica (Sheridan & Stuart, 2018) (Amphibia: Ranidae) from central Vietnam
Source: Biodivers Data J. 2025 Feb 14;13:e145094. doi: 10.3897/BDJ.13.e145094 (PMC11845909; doi:10.3897/BDJ.13.e145094)
Supplement: Supplementary material 1 — Dietary composition of Hylaranaannamitica in Vietnam [file bdj-13-e145094-s001.docx]

**Supplementary files Table 1.** Dietary composition of *Hylarana annamitica* in Vietnam

| Prey category | This study (n = 46) | | | | Trinh et al. (2) (n = 10) | | | |
| --- | --- | --- | --- | --- | --- | --- | --- | --- |
|  | %F | %N | %V | IRI | %F | %N | %V | IRI |
| **Gastropoda** |  |  |  |  |  |  |  |  |
| Pulmonata | 1.14 | 0.29 | 0.31 | 0.58 |  |  |  |  |
| Trochomorphidae | - | - | - | - | 5.26 | 3.45 | 2.87 | 3.86 |
| **Arachnida** |  |  |  |  |  |  |  |  |
| Opiliones | 1.14 | 2.06 | 0.78 | 1.33 | - | - | - | - |
| Araneae | 12.50 | 14.45 | 3.06 | 10.00 | 31.58 | 20.69 | 18.49 | 23.59 |
| **Geophilomorpha** |  |  |  |  |  |  |  |  |
| Geophilidae | 1.14 | 0.29 | 0.52 | 0.65 | - | - | - | - |
| **Blattodea** |  |  |  |  |  |  |  |  |
| Blattidae | 2.27 | 16.81 | 10.53 | 9.87 | - | - | - | - |
| **Coleoptera** |  |  |  |  |  |  |  |  |
| Carabidae | 2.27 | 0.59 | 0.98 | 1.28 | - | - | - | - |
| Cerambycidae | 2.27 | 2.36 | 1.48 | 2.04 | - | - | - | - |
| Curculionidae | 1.14 | 0.59 | 0.02 | 0.58 | 5.26 | 3.45 | 9.94 | 6.22 |
| Elateridae | - | - | - | - | 5.26 | 3.45 | 2.75 | 3.82 |
| Erotylidae | 2.27 | 2.65 | 1.78 | 2.24 | - | - | - | - |
| Eucnemidae | 1.14 | 2.36 | 0.35 | 1.28 | - | - | - | - |
| Lampyridae | - | - | - | - | 5.26 | 3.45 | 8.32 | 5.68 |
| Nosodendridae | 1.14 | 0.29 | 0.05 | 0.49 | - | - | - | - |
| Oedemeridae | - | - | - | - | 5.26 | 3.45 | 1.24 | 3.32 |
| Psephenidae | 1.14 | 0.88 | 0.09 | 0.70 | - | - | - | - |
| Rhysodidae | 1.14 | 0.59 | 0.09 | 0.60 | - | - | - | - |
| Scarabaeidae | 2.27 | 0.59 | 1.04 | 1.30 | - | - | - | - |
| Larvae | 10.23 | 8.26 | 1.53 | 6.67 | - | - | - | - |
| **Dermaptera** |  |  |  |  |  |  |  |  |
| Anisolabididae | 3.41 | 4.13 | 0.29 | 2.61 | - | - | - | - |
| Pygidicranidae | 1.14 | 0.29 | 0.20 | 0.54 | - | - | - | - |
| **Diptera** |  |  |  |  |  |  |  |  |
| Asilidae | 1.14 | 3.24 | 0.92 | 1.77 | - | - | - | - |
| Chironomidae | 1.14 | 0.29 | 0.12 | 0.52 | - | - | - | - |
| Culicidae | 1.14 | 0.29 | 0.08 | 0.50 | - | - | - | - |
| Tipulidae | 1.14 | 1.77 | 0.07 | 0.99 | - | - | - | - |
| **Hemiptera** |  |  |  |  |  |  |  |  |
| Aradidae | 1.14 | 0.29 | 0.08 | 0.51 | - | - | - | - |
| Membracidae | 1.14 | 0.59 | 0.23 | 0.65 | - | - | - | - |
| Nabidae | 1.14 | 0.29 | 0.10 | 0.51 | - | - | - | - |
| Pentatomidae | 1.14 | 0.59 | 5.97 | 2.56 | 15.79 | 10.34 | 10.75 | 12.29 |
| **Hymenoptera** |  |  |  |  |  |  |  |  |
| Dryinidae | 1.14 | 2.36 | 0.33 | 1.27 | - | - | - | - |
| Formicidae | 2.27 | 0.59 | 0.18 | 1.02 | 21.05 | 48.28 | 18.38 | 29.23 |
| Ichneumonidae | 2.27 | 1.18 | 0.21 | 1.22 | - | - | - | - |
| Vespidae | 1.14 | 0.29 | 0.08 | 0.50 | - | - | - | - |
| **Isoptera** |  |  |  |  |  |  |  |  |
| Rhinotermitidae | 3.41 | 3.54 | 0.44 | 2.46 | - | - | - | - |
| **Lepidoptera** |  |  |  |  |  |  |  |  |
| Noctuidae | 1.14 | 2.36 | 13.22 | 5.57 | - | - | - | - |
| Larvae | 7.95 | 3.24 | 3.48 | 4.89 | - | - | - | - |
| Other Lepidoptera | 1.14 | 0.59 | 0.26 | 0.66 | - | - | - | - |
| **Mantodea** |  |  |  |  |  |  |  |  |
| Mantidae | 2.27 | 1.77 | 40.30 | 14.78 | - | - | - | - |
| **Orthoptera** |  |  |  |  |  |  |  |  |
| Acrididae | 7.95 | 10.62 | 2.53 | 7.03 | - | - | - | - |
| Gryllidae | 2.27 | 5.01 | 5.39 | 4.23 | - | - | - | - |
| Tettigoniidae | - | - | - | - | 5.26 | 3.45 | 27.28 | 12.00 |
| **Anura** | 1.14 | 0.59 | 2.02 | 1.25 | - | - | - | - |
| Unidentified | 9.09 | 2.95 | 0.91 | 4.32 | - | - | - | - |
